# Magnetic resonance imaging of the papillary muscles of the left ventricle: normal anatomy, variants, and abnormalities

**DOI:** 10.1186/s13244-019-0761-3

**Published:** 2019-08-19

**Authors:** Prabhakar Rajiah, Nicholas Lim Fulton, Michael Bolen

**Affiliations:** 1 Department of Radiology, Cardiothoracic Imaging, UT Southwestern Medical Center, 5323 Harry Hines Blvd, Dallas, TX 75390 USA; 2 Akron Radiology Inc, 525 E. Market Street, Akron, OH 44304 USA; 3 Imaging Institute, Cleveland Clinic Foundation, 9500 Euclid Avenue, Cleveland, OH 44195 USA

**Keywords:** Papillary muscles, CMR, Variants, Anatomy, Hypertrophic cardiomyopathy

## Abstract

Left ventricular papillary muscles are small myocardial structures that play an important role in the functioning of mitral valve and left ventricle. Typically, there are two groups of papillary muscles, namely the anterolateral and the posteromedial groups. Cardiovascular magnetic resonance (CMR) is a valuable imaging modality in the evaluation of papillary muscles, providing both morphological and functional information. There is a remarkably wide variation in the morphology of papillary muscles. These variations can be asymptomatic or associated with symptoms related to LV outflow tract obstruction, often associated with hypertrophic cardiomyopathy. Abnormalities of the papillary muscles range from congenital disorders to neoplasms. Parachute mitral valve is the most common congenital abnormality of papillary muscles, in which all the chordae insert into a single papillary muscle. Papillary muscles can become dysfunctional, most commonly due to ischemia. Papillary muscle rupture is a major complication of acute myocardial infarction that results in mitral regurgitation and associated with high mortality rates. The most common papillary neoplasm is metastasis, but primary benign and malignant neoplasms can also be seen. In this article, we discuss the role of CMR in the evaluation of papillary muscle anatomy, function, and abnormalities.

**Electronic supplementary material:**

The online version of this article (10.1186/s13244-019-0761-3) contains supplementary material, which is available to authorized users.

## Key points

- The papillary muscles of the left ventricle play an important role in the functioning of the mitral valve.
- The posteromedial papillary muscle has a single arterial supply and the anterolateral muscle has a dual arterial supply.
- Cardiac MRI is an important imaging modality for papillary muscles due to high resolution, multi-planar capabilities, and soft tissue contrast.
- There is a wide variation the morphology of the papillary muscles.
- Papillary muscles abnormalities are common in patients with hypertrophic cardiomyopathy.

## Introduction

The papillary muscles of the left ventricle (LV) are small muscular structures located within the left ventricular cavity. Although small, these muscles play an important role in the functioning of the mitral valve and the left ventricle. Typically, there are two papillary muscles: the anterolateral (AL) and posteromedial (PM) groups. However, there is a remarkably wide variation in papillary muscle morphology. These variations can be asymptomatic or associated with symptoms related to obstruction of the LV outflow tract. Papillary muscle abnormalities range from congenital anomalies to neoplasms. While some of these variations are benign, others are associated with significant morbidity. Papillary muscle dysfunction may result in mitral regurgitation, whereas papillary muscle rupture can be fatal if untreated. Cardiovascular magnetic resonance (CMR) is a modality well suited to the evaluation of papillary muscles, offering good spatial and temporal resolution, inherent soft tissue contrast, and lack of ionizing radiation.

There is only limited information regarding papillary muscles and its imaging in the literature. In this review, we discuss the role of CMR in the evaluation of normal and variant papillary muscle anatomy and in cases of papillary muscle abnormalities.

## Normal papillary muscle anatomy and function

**Fig. 1:**
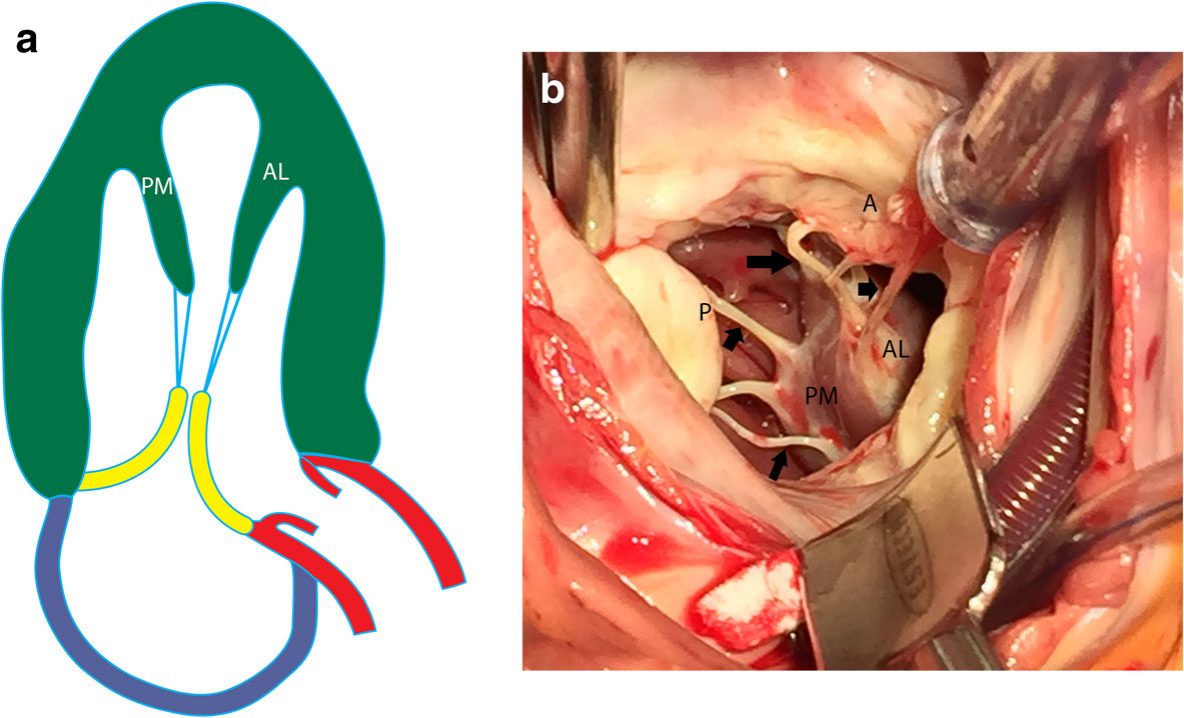
Normal anatomy of papillary muscles. **a** Illustration showing the anterolateral (AL) and posteromedial (PM) in a vertical long-axis projection. The papillary muscles originate from the free wall of the LV attached to trabecula carnea. The papillary muscles give rise to multiple chordae tendinae (blue), which attach to the mitral valve (yellow). **b** Surgical image showing the head of the anterolateral (AL) and posteromedial (PM) muscles attached to their chordae tendinae (arrows), which then attach to the anterior (A) and posterior (P) mitral leaflets

The AL papillary muscle usually originates between the anterolateral and inferolateral walls, whereas the PM muscle originates near the attachment of the inferior wall to the septum (Fig. 1a) [1]. The attachment of the papillary muscle to the LV wall can be “finger-like,” with a small focal point of attachment and few or no trabecular attachments, or “tethered,” with a large base of attachment and several trabecular bridges [2]. However, recent cross-sectional studies [3] have shown that most papillary muscles do not attach directly to the LV wall but instead attach to a network of trabeculae carneae, elongated strands of muscles lining the LV that attach to the solid portion of the LV wall [3]. Each papillary muscle has a major trunk containing approximately six projections (or heads) (Fig. 1b) [4]. The papillary muscle head contains approximately 12 chordae tendinae (connective tissue strands), each of which subdivides into two secondary chordae tendinae, which subdivide into two or three tertiary chordae tendinae. Ultimately, each individual papillary muscle is affiliated with an average of 62 chordae. The chordae tendinae are attached to the tips of both mitral valve leaflets; hence, damage to one papillary muscle may affect both leaflets [4].

The PM muscle has a single arterial supply in the majority of population (63 %), typically by the right coronary artery (posterior descending branch) in a right-dominant system and by the left circumflex artery (LCX) (typically the 3rd obtuse marginal branch) in a left-dominant system [5]. AL papillary muscle has a dual blood supply in 71% of patients, both from LCX (first obtuse marginal branch) and LAD (1st diagonal branch), providing increased protection against ischemia. The papillary muscles are the last portion of the heart to be perfused and are therefore are at high risk for ischemia [4]. Papillary muscles are supplied by channels that originate from the epicardial vessels that extend radially inward and supply in a segmental distribution i.e., separate branches are received for basal, mid-portion, and tips of the muscles [6].

The papillary muscles play an important role in mitral valve function. During systole, the papillary muscles contract before LV wall contraction [7], which results in apposition of the mitral valve leaflets, limiting the retrograde flow of blood from the LV back into the left atrium. If an ectopic ventricular beat results in contraction of the LV wall before the papillary muscles, an element of mitral regurgitation will be present.

## Imaging of papillary muscles

Echocardiography is the first-line imaging modality used in the evaluation of cardiac abnormalities, including papillary muscles. On transthoracic echocardiograms, papillary muscles are well demonstrated in the short-axis view [8], 1–3 cm apical to the level of the mitral valve laterally [9]. Echocardiography allows for real time cine depiction of papillary muscle anatomy and function. This is obtained through a combination of 2D and 3D grey scale imaging for morphologic deepiction, with Doppler imaging providing additional hemodynamic insights. Limitations of echocardiogram include operator dependence, limited soft tissue contrast, and limited field-of-view in a range of scenarios based upon patient anatomy (COPD, obesity, narrow rib spacing), mobility, as well as post operative status with overlying hardware and bandages [10]. Computed tomography (CT) can be used in the evaluation of papillary muscle as well as other components of mitral valve apparatus. The high isotropic spatial resolution of CT allows exquisite morphological delineation and accurate measurement, which is essential for surgical and interventional techniques. With retrospective ECG-gating, dynamic evaluation of the papillary muscles and quantification of ventricular function can be performed, however there is limited assessment of flow dynamics. While CT imaging is associated with ionizing radiation, this can be minimized with dose reduction strategies such as tube current modulation, as well as lower tube voltage and current settings [11, 12].

## CMR of papillary muscles

CMR is well suited to the evaluation of papillary muscles because of its good spatial and temporal resolution, wide field-of-view, and multi-planar imaging capabilities. CMR has high intrinsic soft tissue contrast that can be amplified with gadolinium-based contrast agents, which may lead to improved tissue characterization. A balanced steady-state free precession (b-SSFP) sequence is used to evaluate the anatomy and function of papillary muscles, ventricles, and valves. Dynamic evaluation of papillary muscle contraction as well as measurement of muscle thickness and mass can be performed [13]. Novel 3D-cine-SSFP sequences enable 4D reconstruction, which allows for dynamic evaluation of papillary muscle motion in any plane. Papillary muscle contraction and dynamic motion can be evaluated objectively with myocardial tagging techniques; deformation (or “strain”) of the tag lines can be quantified as a percentage, signifying the amount of contraction that occurs in systole [14, 15] (Additional file 1: Movie S1). Contrast enhancement can be evaluated in the early phase with dynamic first-pass perfusion images, T1-weighted images, or late gadolinium-enhanced (LGE) images obtained 10 to 15 min after contrast administration. LGE is useful in cases of cardiomyopathy, infarction, and masses. T1- and T2-weighted dark blood sequences with and without fat saturation are useful to in characterization of masses in papillary muscles. Parametric techniques, such as T1, T2, and extracellular volume (ECV) mapping are also useful in tissue characterization and quantification.

## Normal appearance of papillary muscles on CMR

**Fig. 2:**
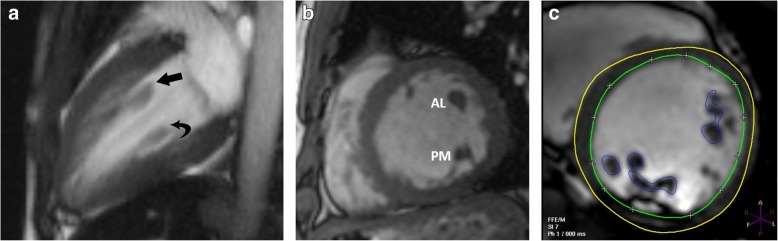
MRI appearances of normal papillary muscles. **a** Two-chamber vertical long-axis SSFP MRI through the left ventricle shows the anterolateral (straight arrow) and posteromedial (curved arrow) papillary muscles. **b** Short-axis SSFP MRI image shows the anterolateral (AL) and posteromedial (PM) papillary muscles. **c** Short-axis SSFP MRI image shows the technique for measuring papillary muscle mass. The papillary muscles have been contoured (blue) in end-diastolic image to derive papillary muscle mass. The endocardial (green) and epicardial (yellow) contours are also seen

Papillary muscles have the same signal intensity as the myocardium in all sequences: intermediate on T1-weighted, T2-weighted, and b-SSFP sequences. Early and late contrast enhancement patterns are also similar to those of normal myocardium. The axis of the papillary muscles is oriented parallel to the axis of the LV (Fig. 2a) and perpendicular to the mitral annulus (Fig. 2b); the length of the papillary muscles is variable. The thickness of the papillary muscles is approximately equal to the thickness of the septal or left ventricular free wall; however, the AL muscle tends to be slightly thicker than the PM muscle. Papillary muscle mass measured in end-diastole (Fig. 2c) is normally higher in men than in women. Papillary muscle mass accounts for 8.9 ± 1.4% of LV mass, and there is a significant correlation between papillary and LV wall mass (*r* = 0.81; *P* < .001). Papillary muscle mass also correlates with body surface area, but there is weak or no correlation with age, body mass index, weight, and height [16–19].

## Papillary muscle variants

**Fig. 3:**
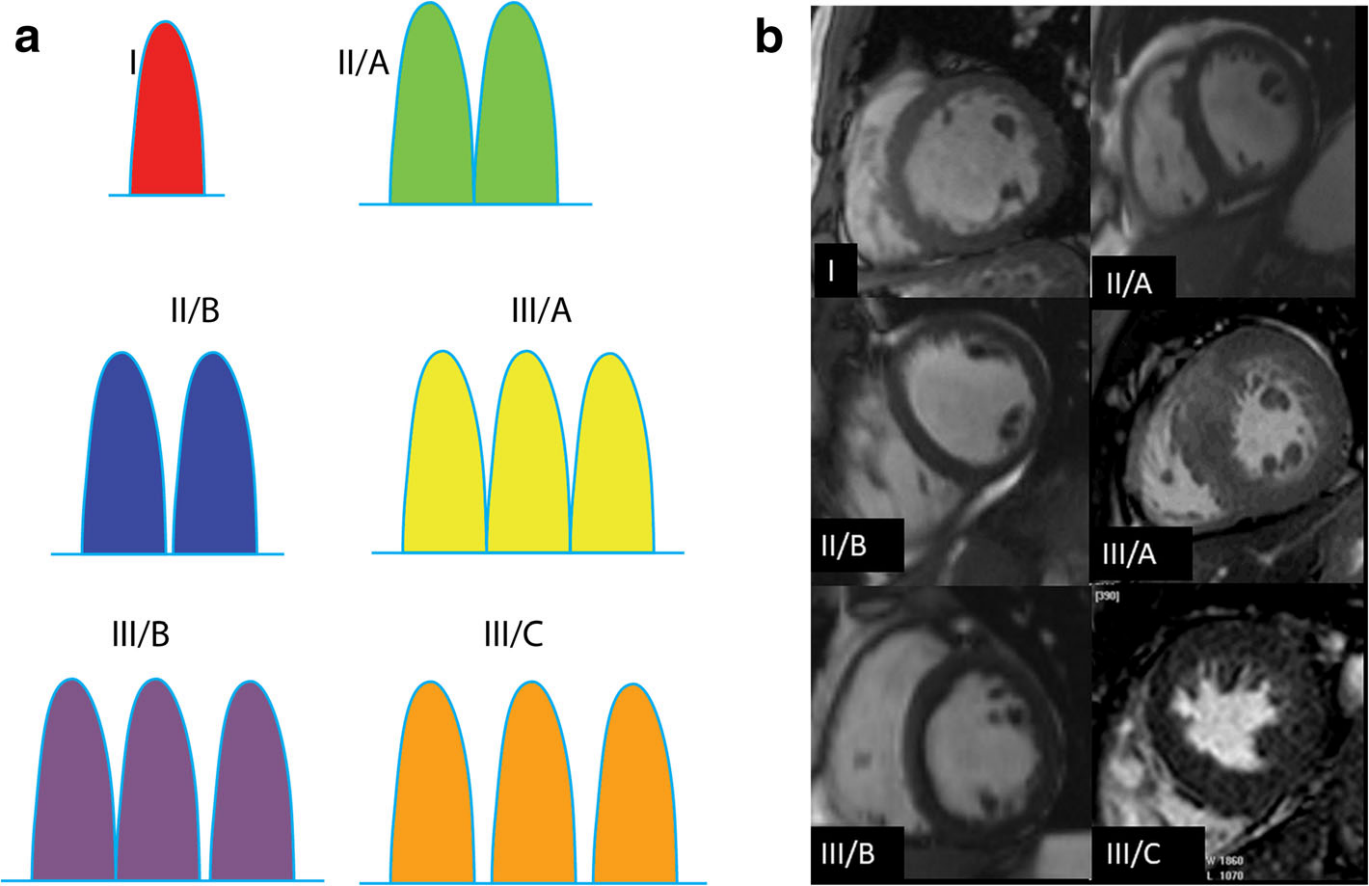
Papillary muscle variations. **a** Illustration of six papillary muscle variations. Type I is a single muscle, type II/A is two heads with a common origin, type II/B is two heads separated at the basal portion, type III/A is three heads with a common origin, type III/B is three heads with two sharing a common origin, and type III/C is three heads with no common origin. **b** Multiple short-axis SSFP MRI images demonstrating each of the papillary muscle variations

There is significant variability in papillary muscle morphology (Fig. 3). Papillary muscles can be categorized by the number of muscle heads and then subcategorized depending on whether the heads share a common basal segment or have unique basal segments [20]. Typically, the AL muscle has a single major muscle group, whereas the PM muscle contains two or three major muscle groups [4]. When multiple muscle groups are present, they may share a common origin or may have separate origins. Although many of these variants are considered normal and incidental, some papillary muscle variants result in functional deficits contributing to complications such as left ventricular outflow obstruction. The significant variations include anomalous insertion, accessory muscles, antero-apical displacement, double bifid morphology, and hypermobile muscles, which are discussed in detail in the section on obstructive lesions.

## Congenital abnormalities

### Parachute mitral valve

**Fig. 4:**
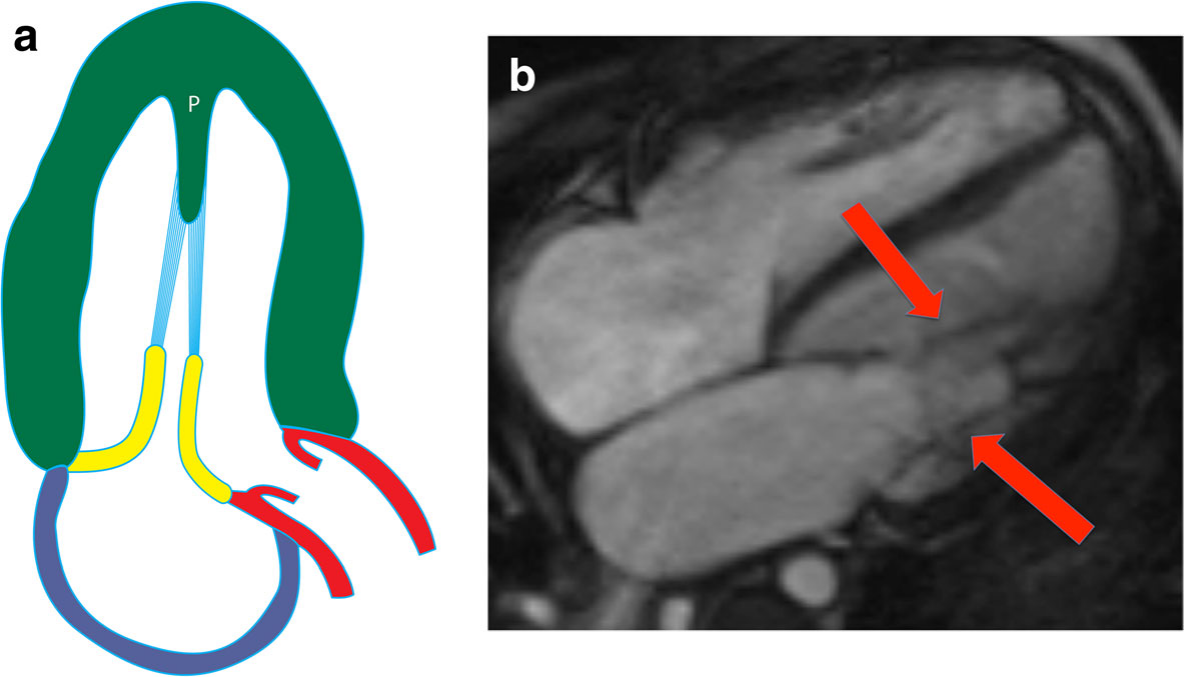
Parachute mitral valve. **a** Illustration of a parachute mitral valve. The chordae tendinae (blue) originate from a single papillary muscle (P). **b** Four-chamber SSFP image of parachute mitral valve, with all of the chordae (arrows) arising from a single papillary muscle

Parachute mitral valve is a rare congenital anomaly in which all of the chordae tendinae originate from a  single papillary muscle [21, 22] (Fig. 4a). The common insertion is into the PM muscle in three fourths of cases. The mitral valve represents a “parachute,” the papillary muscle is the “skydiver,” and the chordae tendinae are the “strings” of the parachute connecting the two. Because of the abnormal chordal attachment, the mitral valve is shaped like a funnel in these cases. The chordae are short and thickened, limiting the movement of the mitral valve cusps and resulting in stenosis.

Parachute mitral valve is the most common cause of isolated mitral stenosis in children [23]. Mitral incompetency or normal valve function can also be seen. Parachute mitral valve is also seen in association with other congenital cardiac abnormalities, especially ventricular septal defects, valvular aortic stenosis, pulmonic stenosis, and patent ductus arteriosus [24]. Although this condition usually presents during childhood, milder lesions may present in adults as dyspnea, overt cardiac failure, pulmonary hypertension, and recurrent infections.

On CMR images of parachute mitral valve, all of the chordae tendinae arising from the mitral valve leaflets attach to a single papillary muscle (Fig. 4b). In diastole, a jet of dephasing spins is often seen passing from the left atrium to the LV due to mitral valvular stenosis. Occasionally, mitral incompetence may also be seen. In severe cases, mitral valvotomy or mitral valve replacement may be necessary, particularly in cases of subaortic stenosis. Findings such as LV hypoplasia and atrial septal defects are associated with poorer postoperative outcomes [25].

### Parachute-like mitral valve

**Fig. 5:**
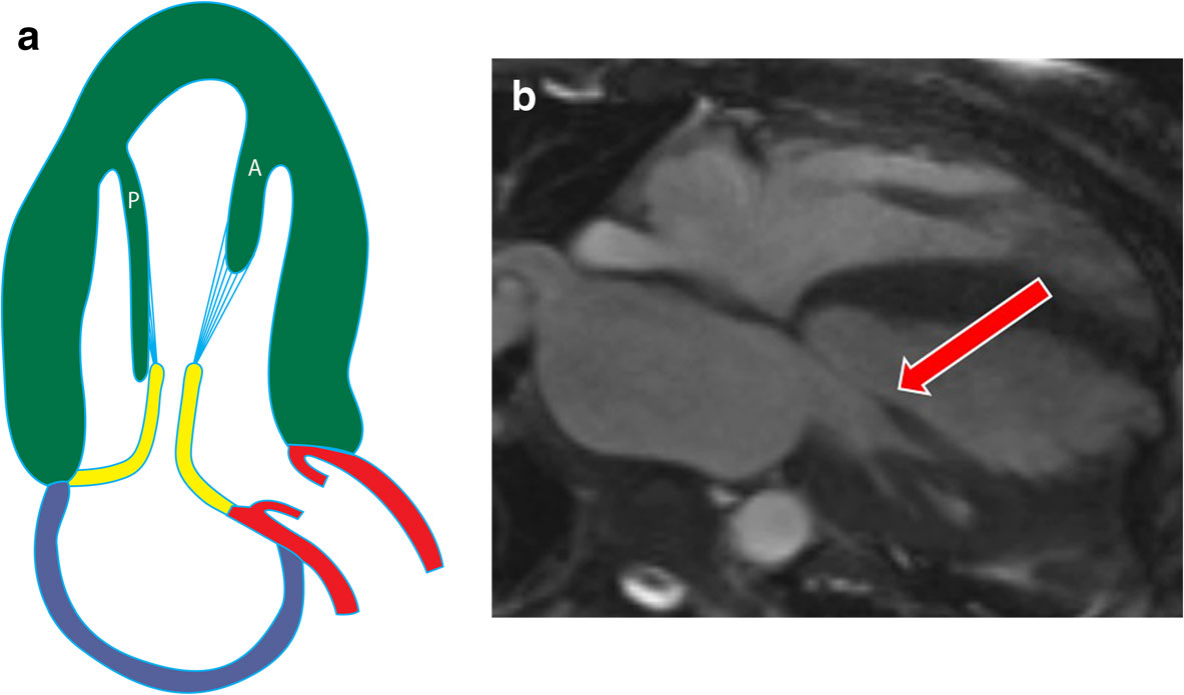
Parachute-like asymmetric mitral valve. **a** Illustration showing the posteromedial papillary muscle (P) is longer and attaches more proximally to the mitral valve, compared to the anterolateral (A) papillary muscle. **b** Four-chamber cine SSFP image shows asymmetric elongation of a single papillary muscle (anterolateral muscle) (arrow) attaching to the mitral valve leaflets, resulting in an eccentric mitral orifice

Parachute-like asymmetric mitral valve is more common than parachute mitral valve (Fig. 5a). In individuals with this condition, there are two papillary muscles present. One of the papillary muscles is normal and the other is elongated with its tip attached to the mitral valve. The abnormal papillary muscle has few or no chords, resulting in an eccentric mitral valve orifice [25]. It is controversial whether parachute-like asymmetric mitral valve should be considered a variant of parachute mitral valve, as their developmental etiologies differ [25].

On CMR images of the parachute-like mitral valve, both of the papillary muscles are present, but one is elongated and located asymmetrically higher in the LV (Fig. 5b). The attachment of the papillary muscles at the mitral valve is also asymmetric, resulting in an asymmetric mitral valve orifice (Additional file 2: Movie S2). Unlike true parachute mitral valves, parachute-like asymmetric mitral valves may be asymptomatic. However, case reports have suggested an increased propensity toward the development of infective endocarditis in patients with this condition because of turbulent flow across the abnormal valve [26].

### Shone complex

**Fig. 6:**
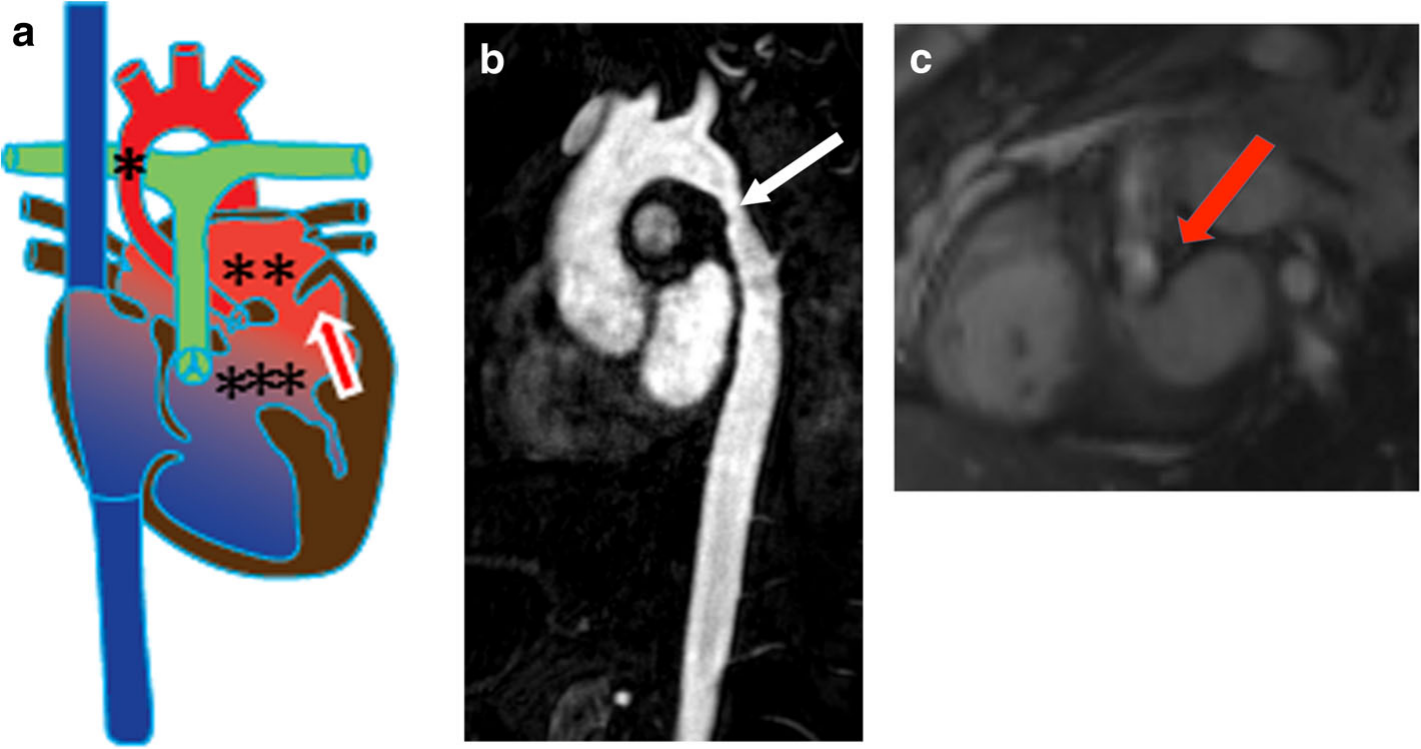
Shone complex. **a** Illustration of Shone complex showing aortic coarctation (*), supravalvular mitral ring (**), subaortic stenosis (***) and a parachute mitral valve (arrow). **b** Sagittal MRA of the aorta shows coarctation (arrow). **c** Short-axis cine SSFP image at the level of LVOT depicting a subaortic stenosis (arrow) with flow acceleration seen at the subaortic level

Shone complex, or Shone’s anomaly, is a tetralogy of four lesions: parachute mitral valve (described above), aortic coarctation, supra-valvular mitral ring, and subaortic stenosis (Fig. 6a). Nearly two thirds of patients present with all four lesions, but the presence of any three of these lesions is sufficient for the diagnosis of Shone complex. Aortic coarctation can be easily identified on CMR as a focal narrowing of the thoracic aorta best demonstrated on sagittal or left anterior oblique views (Fig. 6b) [27, 28]. A supra-valvular mitral ring appears as a membrane within the left atrium above the mitral valve orifice. This ring may attach to the mitral valve and restrict its mobility or may directly obstruct blood flow through the mitral valve [29]. Subaortic stenosis in Shone complex can take two forms, muscular (Fig. 6c) or membranous [30]. Surgical management of this condition includes correction of the coarctation, resection of the lesion causing subaortic stenosis, and possible LV outflow tract (LVOT) reconstruction or bypass. When applicable, associated lesions such as ventricular septal defects may also require repair [27].

### Double-chambered LV

A double-chambered LV is a rare congenital anomaly in which the LV is divided by an abnormal musculature/septum between the mitral valve and papillary muscles [31, 32]. There is a wide communication between this abnormal outpouching and the cardiac chamber. Most cases are congenital due to mal-development of the myocardial intra-trabecular sinusoids; this condition can also be associated with endocardial fibroelastosis or hypertrophic cardiomyopathy (HCM) [32]. A double-chambered LV is asymptomatic and often an incidental finding.

On CMR, the abnormal muscular band/septum can be seen dividing the LV into two chambers (Additional file 3: Movie S3 and Additional file 4: Movie S4). This band extends across the LV, connecting the papillary muscles. Dynamic imaging will demonstrate that the double-chambered LV has a normal contraction in systole, thus distinguishing this entity from an aneurysm [33]. Additionally, double-chambered LV does not demonstrate LGE. Surgical treatment can be performed if needed (typically when this condition is associated with other cardiac abnormalities).

### LV non-compaction

LV non-compaction is a type of cardiomyopathy in which there is an exaggerated component of non-compacted LV myocardium. This is caused by persistence of primitive embryonal cardiac sinusoids beyond fetal life, with a failure to mature to compacted myocardium. On CMR images of LV non-compaction, increased trabeculation can be seen in the LV. One study suggested that an end-diastolic ratio of non-compacted to compacted myocardium of > 2.3 is diagnostic for LV non-compaction (sensitivity, 86%; specificity, 99%) [34] (Additional file 5: Movie S5). However, a more recent study [35] demonstrated the lack of specificity of this finding. Additional findings such as an abrupt transition between normal and thinned myocardium in the non-compacted area may increase the specificity of this technique. Complications of LV non-compaction include ventricular arrhythmia, thromboembolism, and ventricular dysfunction. LGE in the trabeculations is seen in 40% of cases [36] and is a predictor of poor prognosis.

## Papillary muscle thickening

**Fig. 7:**
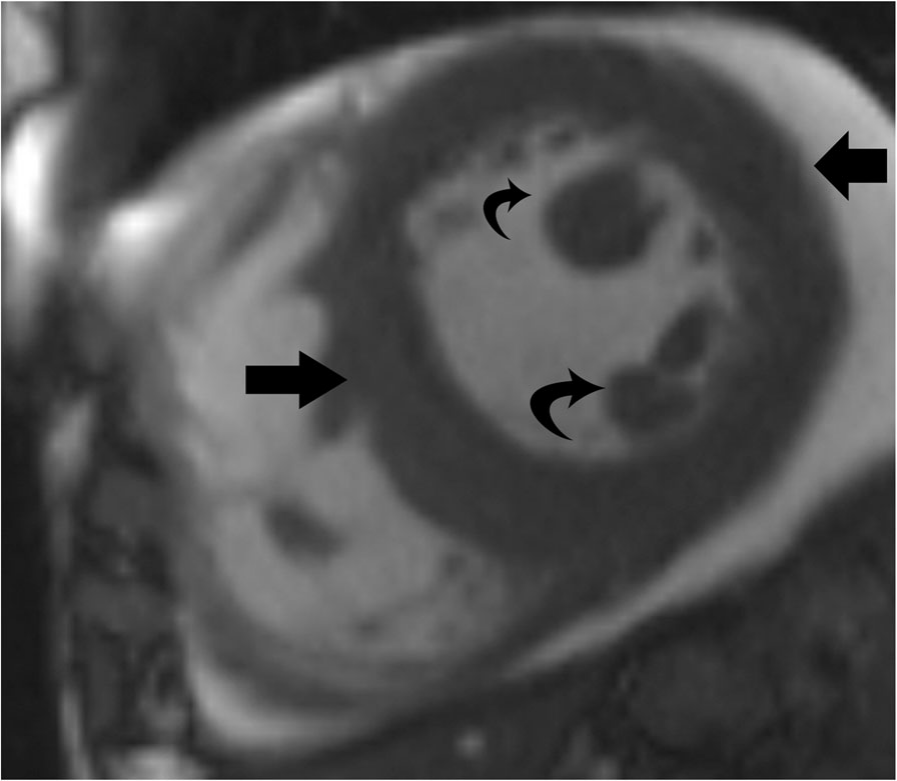
Papillary muscle hypertrophy. Short-axis cine-SSFP image in a patient with systemic hypertension shows hypertrophy of the papillary muscles (curved arrow), which is proportionate to the concentric LV hypertrophy (straight arrows)

Papillary muscle thickening can be caused by hypertrophy or infiltrative disorders (e.g., amyloidosis, sarcoidosis, iron deposition) and may involve one or both of the papillary muscles. Common causes of hypertrophy include systemic hypertension and HCM. In cases of hypertension, there is proportional hypertrophy of the papillary muscles (increased thickness and mass) along with concentric LV hypertrophy [4] (Fig. 7). Proportionate thickening is also typically seen in cases of the concentric type of HCM. On CMR images, papillary muscle thickness > 1.1 cm or greater than the LV free wall thickness is abnormal

## Obstructive lesions

### Hypertrophic cardiomyopathy

**Fig. 8:**
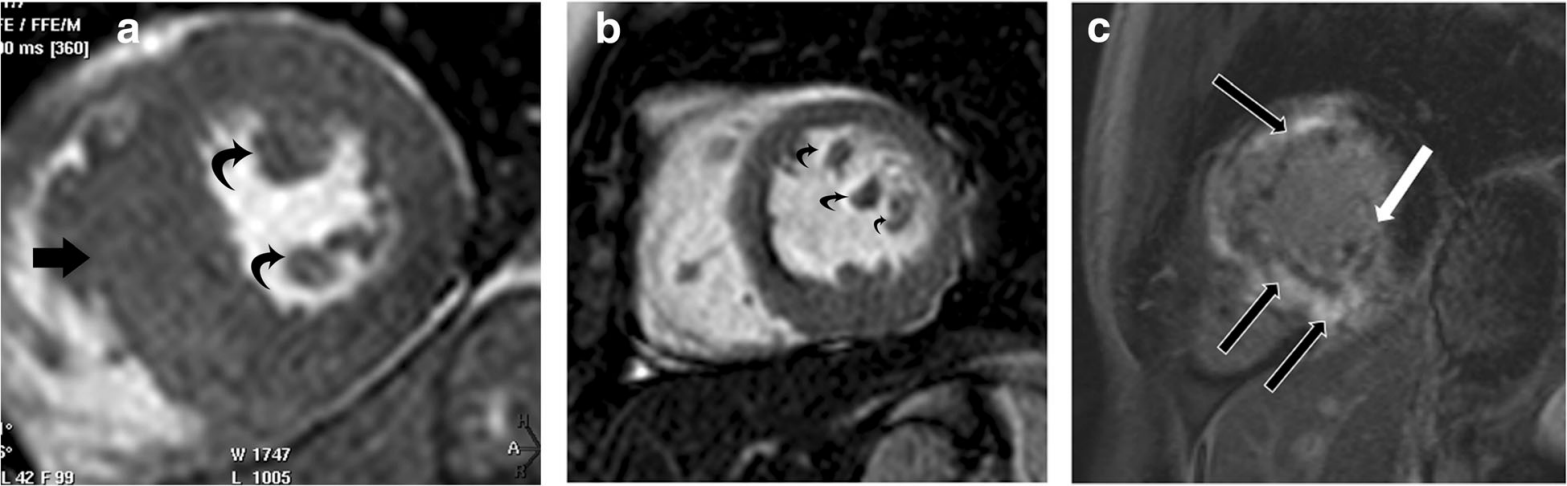
Hypertrophic cardiomyopathy. **a** Short-axis view SSFP image shows severe hypertrophy of the ventricular septum (straight arrow) and hypertrophy of the papillary muscles (curved arrows). **b** Short-axis SSFP image in another patient shows 3 papillary muscles which are hypertrophied (curved arrows). **c** Short-axis view delayed-enhancement image shows extensive mid-myocardial delayed enhancement (black arrows) due to interstitial fibrosis in hypertrophic cardiomyopathy. The posteromedial papillary muscle also shows patchy areas of delayed enhancement (white arrow)

HCM represents a diverse collection of abnormalities. The most common phenotype of HCM is asymmetric septal hypertrophy, followed by mid-ventricular, apical, concentric, and mass-like subtypes. On CMR, hypertrophy of the myocardium ( > 11 mm) in different patterns is seen [37] (Fig. 8). LGE due to interstitial fibrosis may also be seen in hypertrophied and non-hypertrophied segments, typically in a mid-myocardial, patchy distribution, and often at right ventricle (RV) insertion points (Fig. 8c). LGE is associated with worse symptoms, more severe cardiac dysfunction, and the development of ventricular dysrhythmias [37, 38].

In patients with HCM, the papillary muscles are frequently hypertrophied, with a mass measuring roughly twice the mass of papillary muscles in healthy controls; 54% of papillary muscles in these patients have a mass > 7 g/m^2^ (> 2 standard deviations above normal), and 20% of patients have severe hypertrophy [39] (Fig. 8a). The hypertrophy of papillary muscles correlates with LV wall thickness and myocardial mass [2], and the papillary muscle mass index is also weakly correlated with the magnitude of outflow gradient [2]. These findings suggest that papillary muscle hypertrophy may not be caused by a primary genetic abnormality alone but may also be secondary to LV pressure overload from LV obstruction [39]. The distance between the papillary muscle and the septum is also a point of interest in patients with HCM, as this distance was noted to be smaller in patients with obstruction [39]. In addition, patients with HCM also tend to have a larger number of papillary muscles (2.5 muscles vs 2.1 muscles in controls), with half of the patients demonstrating multiple (i.e., 3 or 4) papillary muscles (Fig. 8b) [39].

On CMR, patchy areas of LGE may be seen in the papillary muscles due to interstitial fibrosis in 6% of patients with HCM [21, 39] (Fig. 8c); these patients tend to have higher papillary muscle mass than patients without LGE [39].

## Solitary papillary muscle hypertrophy

**Fig. 9:**
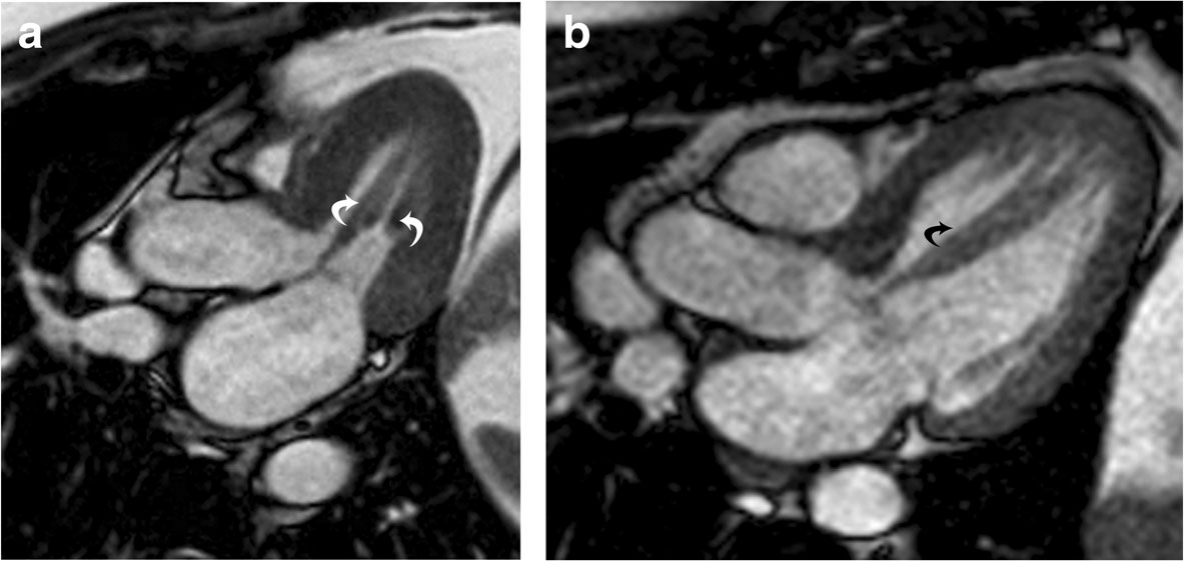
Solitary papillary muscle hypertrophy. **a** Three-chamber SSFP image shows significant hypertrophy of the papillary muscles (curved arrows), without significant myocardial hypertrophy, which is causing narrowing of the mid-ventricular cavity in systole. **b** Three-chamber SSFP image in another patient shows hypertrophy of the anterolateral papillary muscle (curved arrow) with normal thickness of the LV myocardium

Solitary papillary muscle hypertrophy is a unique phenotype of HCM that is seen in 19 to 20% of patients. In this condition, the papillary muscles are hypertrophied but the rest of the left ventricular myocardium is spared [39, 40]. These patients present with angina, dyspnea, syncope, and sudden cardiac death [40]. On CMR images, hypertrophic papillary muscles may demonstrate dynamic mid-cavity obstruction and flow acceleration within the LV (Fig. 9, Additional file 6: Movie S6). The inexperienced viewer may easily overlook a papillary muscle abnormality in the absence of LVH and mistake a case of HCM for a normal examination.

### Anomalous insertion of papillary muscle

**Fig. 10:**
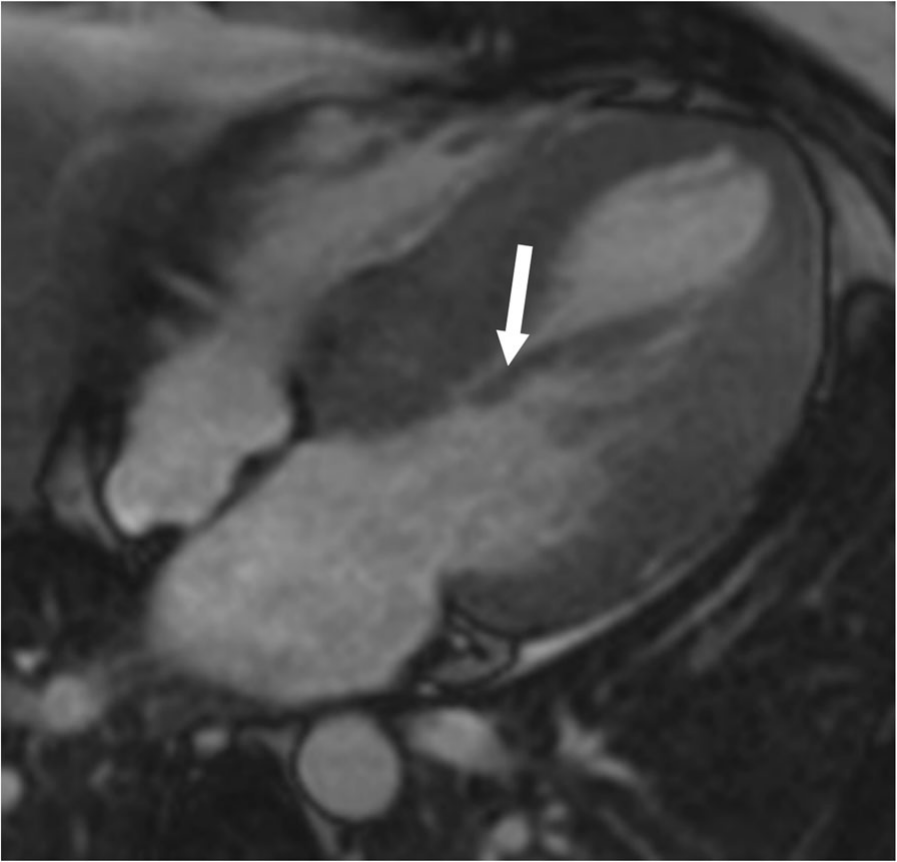
Anomalous papillary muscle insertion. Anomalous direct insertion of the anterolateral papillary muscle to the anterior mitral leaflet (arrow) without intervening chorda tendinae

**Fig. 11:**
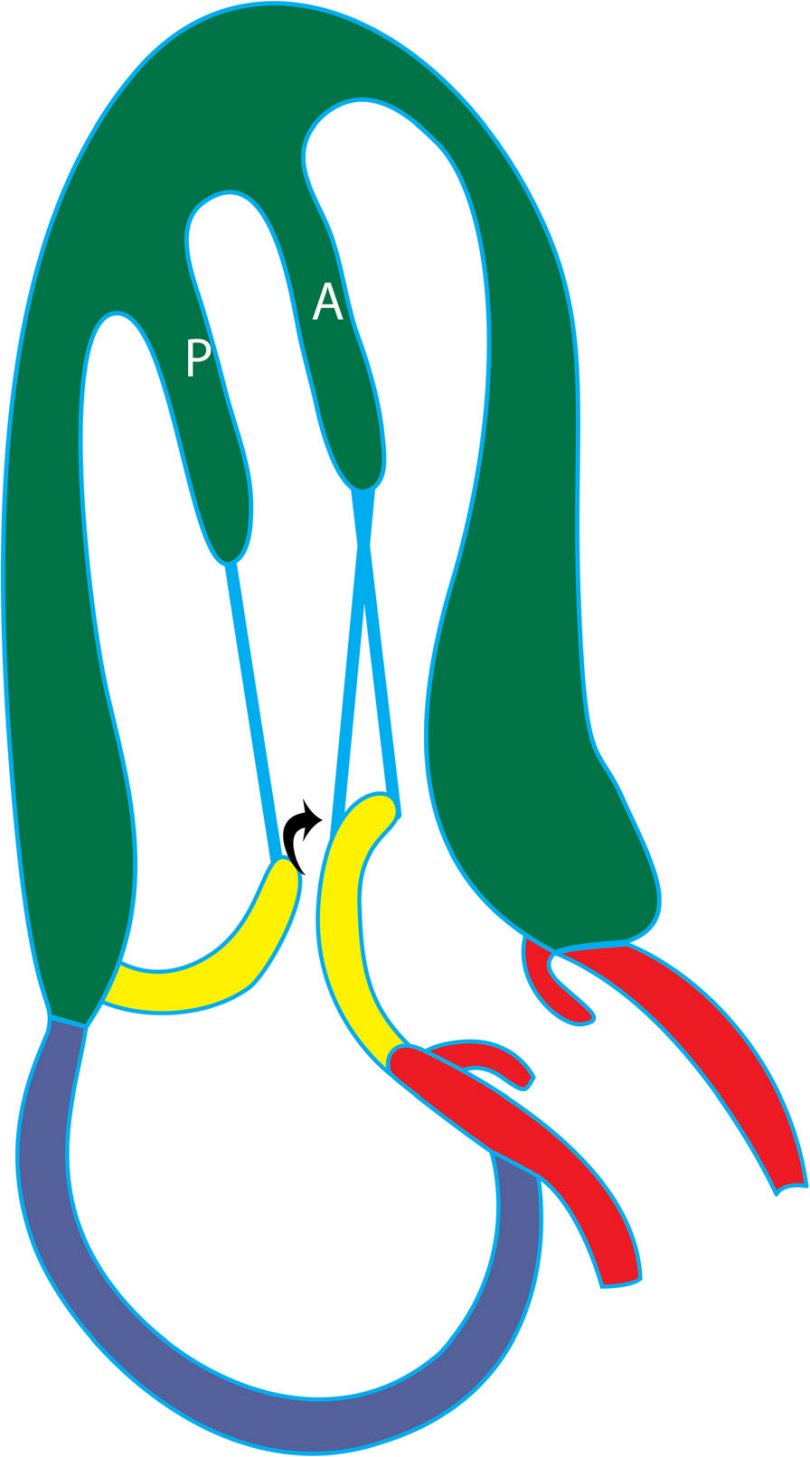
Anomalous papillary muscle insertion. Illustration of anomalous insertion of the anterolateral papillary muscle (A) into the mid-portion of anterior mitral leaflet (arrow), which results in leaflet slack and narrowing of the LVOT

Anomalous insertion of the papillary muscles is a common variant seen in the setting of HCM, occurring in approximately 10% of cases [41]. In one type of anomalous insertion, the papillary muscles bypass the usual connection of the chordae tendinae and insert directly into the mitral valves (Fig. 10). In another type, the chordae tendinae insert into the mid or basal portions of the mitral leaflets instead of into the tips (Fig. 11, Additional file 7: Movie S7). When the papillary muscles attach in an abnormal fashion, they occupy an atypical location within the LV, which may result in dynamic mid-cavity obstruction.

### Accessory papillary muscles

The apical-basal muscle bundle refers to an accessory papillary muscle or muscle bundle that extends from the LV apex and inserts into the basal myocardium without insertion into the mitral leaflet. This condition is commonly present in patients with HCM (63%) and in gene-positive family members (60% vs 10% of controls). On CMR, an accessory papillary muscle is best visualized on the 3-chamber view as a single band of muscle extending from the LV apex through the LV cavity to the basal anteroseptum or anterior wall (Additional file 8: Movie S8). This accessory muscle is typically not associated with LVOT obstruction and is independent of septal thickness. However, it is an important morphological marker for HCM and an independent and primary component of HCM disease expression; this muscle may be useful for screening family members for HCM [42].

### Anteroapical displacement of papillary muscles

**Fig. 12:**
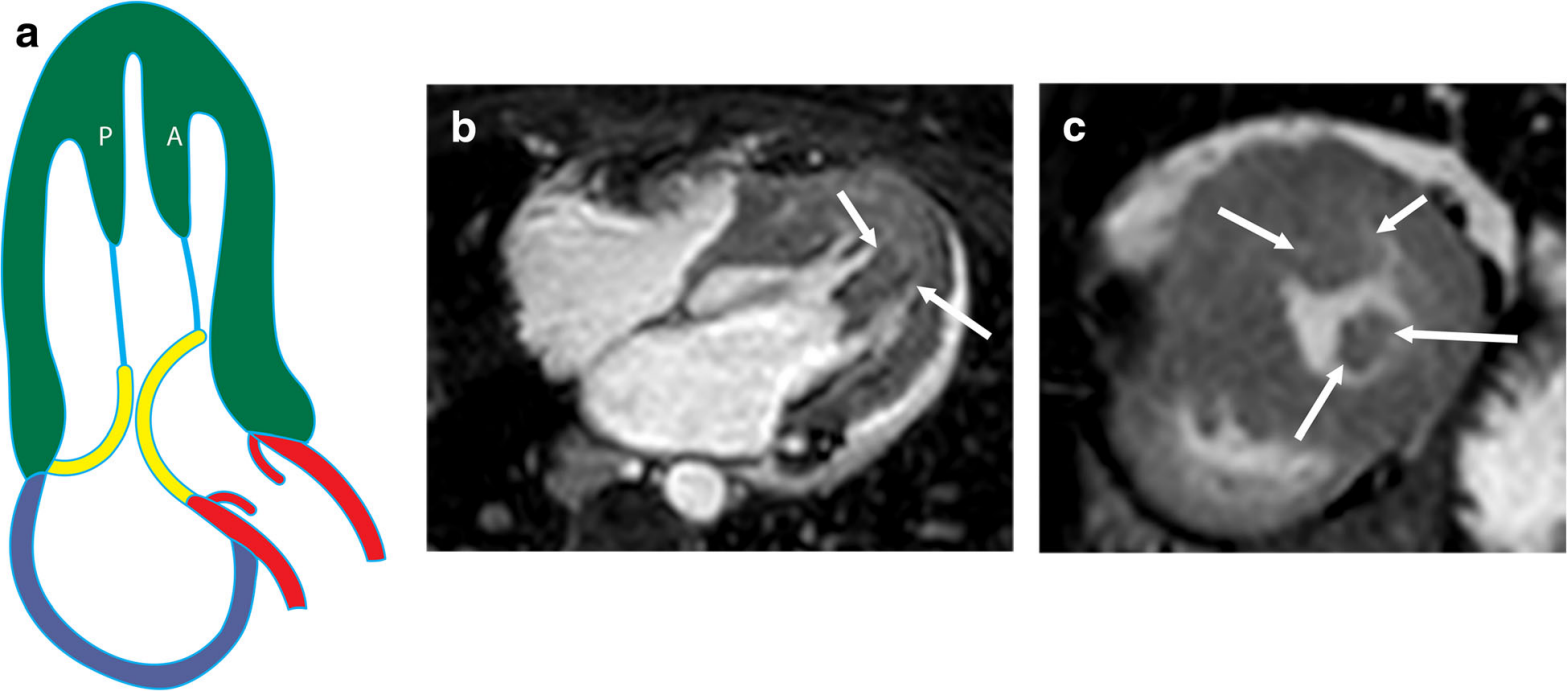
Anteroapical displacement. **a** Illustration showing apical displacement of the papillary muscles (A and P) resulting in leaflet slack and left ventricular outflow tract obstruction during systole. **b** Four-chamber cine 3D-SSFP image shows anteroapical displacement of hypertrophied bifid papillary muscles (arrow) in a patient with HCM. **c** Short-axis SSFP MRI image at the apical level shows the presence of double bifid papillary muscles (arrows), indicating anteroapical displacement

Anterior and apical displacement of the base of the papillary muscle is more common in patients with HCM than in healthy controls (77% vs 17%) and usually involves the AL muscle [43]. This displacement results in leaflet slack; the mitral valve subsequently moves toward the septum, resulting in systolic anterior motion of the mitral valve and causing LVOT obstruction [44]). This abnormality is associated with higher LVOT gradients and systolic anterior motion of the anterior mitral valve leaflet**,** independent of septal thickness or medical therapy (Fig. 12a) [43]. On CMR images of patients with this condition, the base of the papillary muscle is displaced anteriorly (in relation to the ventricular septum) and distally in 2- or 4-chamber views (Fig. 12b). The papillary muscle is also visible in the distal-most apical short-axis image (Fig. 12c).

### Bifid papillary muscles

**Fig. 13:**
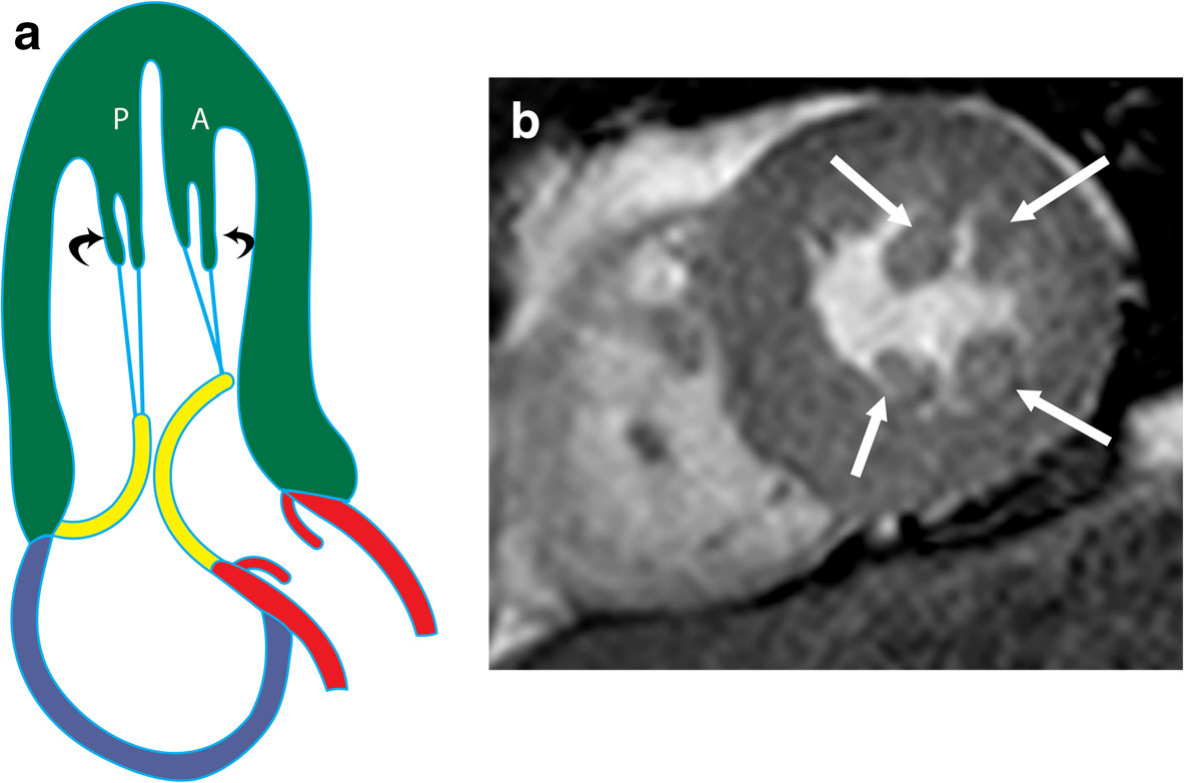
**a** Illustration shows double bifid papillary muscles (curved arrows), which causes systolic LVOT obstruction due to leaflet slack. **b** Short-axis MRI image shows double bifid papillary muscles (arrows)

Bifid papillary muscles are characterized by the presence of more than one muscle head. This may be seen in one or both papillary muscle groups (double bifid morphology) (Fig. 13a). Double bifid papillary muscles are more common in patients with HCM than in healthy controls (70% vs 17%) [43]. This morphology may result in leaflet slack/tethering, systolic anterior motion of the mitral valve, and LVOT obstruction [43, 44]. Bifid papillary muscles are often hypermobile [45] and may be associated with premature ventricular complexes resulting in bigeminy, even in the absence of obstruction [46]. On CMR imaging of bifid papillary muscles, double papillary heads can be seen on multiple cine images (Fig. 13b).

### Hypermobile papillary muscles

Hypermobile papillary muscles with normal morphology and attachment may be the sole manifestation of HCM in some patients [47]. Hypermobility is independent of the underlying degree of septal thickening. This hypermobility can result in mid-cavity obstruction of the LV. On CMR images, hypermobility of the papillary muscles may be associated with septal contact, systolic anterior motion of the mitral valve, LVOT narrowing, and obstruction with LVOT gradients (Additional file 9: Movie S9). Occasionally, hypermobility is not seen on routine cine-MR images; such cases may require the administration of amyl nitrite to allow for identification on imaging [48].

### Elongated anterior mitral leaflet

**Fig. 14:**
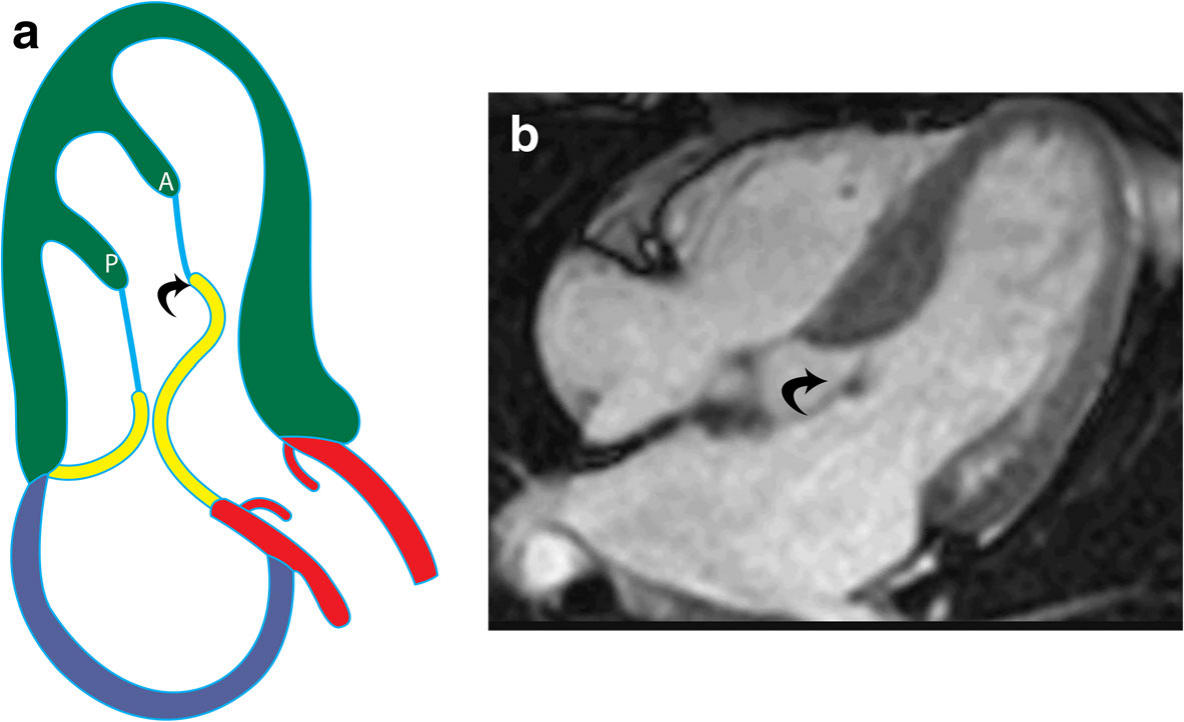
Elongated mitral leaflet. **a** Illustration of elongated mitral valve leaflet (curved arrow), resulting in systolic obstruction of the left ventricular outflow tract, without evidence of other abnormalities related to hypertrophic cardiomyopathy. **b** Four-chamber cine SSFP image shows an elongated anterior mitral leaflet (curved arrow), which causes LVOT obstruction

Elongation of the anterior mitral valve leaflet can contribute to leaflet slack (Fig. 14a) even in the absence of LV or papillary muscle hypertrophy. This is more commonly seen in patients with HCM than in healthy controls, independent of demographic and clinical variables [41, 49]. In 30% of patients with HCM, the elongation is > 2 standard deviations above the length of controls [49]. This condition is associated with SAM and subsequent LVOT obstruction due to leaflet hypermobility (Additional file 10: Movie S10). If both mitral valve leaflets are elongated, leaflet coaptation may be sufficient to avoid significant mitral insufficiency [50]. CMR can play an important role in the detection and characterization of these elongated mitral valve leaflets and can also aid in preoperative planning by providing a roadmap for the surgeon [45, 51–54].

## Miscellaneous abnormalities

### Papillary muscle dysfunction

Papillary muscle dysfunction with a morphologically normal muscle is usually an acquired condition that results in mitral regurgitation. Common causes are transient ischemia, LV dilation, fibrosis in the adjacent LV free wall, or a small LV cavity [4]. Transient ischemia is the most common cause, producing mitral regurgitation during an anginal attack. With LV dilation, the remodeling causes migration of the LV wall caudolaterally from the mitral annulus, resulting in the oblique orientation of the papillary muscles with secondary dysfunction and regurgitation. Scarring/aneurysm of the LV adjacent to the papillary muscle causes dysfunction due to abnormal papillary muscle anchoring, which is caused by the abnormal LV motion. In patients with HCM, the AL papillary muscle may be distorted by thickening of the mid-septum, preventing proper contraction in systole.

On CMR images, the abovementioned etiologies are evident. The resulting mitral regurgitation is usually eccentric (Additional file 11: Movie S11 and Additional file 12: Movie S12) [55], whereas other etiologies such as rheumatic heart disease tend to demonstrate a central jet [56]. Fibrosis of the papillary muscle or the LV wall may also be seen. In chronic ischemic mitral regurgitation, operative repair is usually successful in diminishing the extent of regurgitation and improving symptoms. Mitral annuloplasty and papillary muscle relocation are the surgical options [57, 58].


Additional file 11:**Movie S11. 11. 4** chamber SSFP cine clip in a patient with myocardial infarction and LV dilation shows, a posteriorly directed mitral regurgitation as a consequence of LV dilation. (MP4 383 kb)


### Papillary muscle fibrosis

Papillary muscle fibrosis/necrosis without rupture is common, as the papillary muscles are the last portion of the heart to be perfused. Coronary atherosclerosis is the most common cause, but this condition can also be caused by shock, infective endocarditis, acute valvar regurgitation, anemia, LVOT obstruction, systemic hypertension, cardiomyopathies, endocardial fibroelastosis, endomyocardial fibrosis, myocardial disorders, and anomalous origin of the coronary arteries from the pulmonary arteries [4]. Fibrosis can involve one or both of the papillary muscles but is more common in the PM muscle, likely due to the more common single vascular supply. Pathologically, there is either an overgrowth of class A vessels or an interruption of all channels [6]. Mitral regurgitation can occur in patients with papillary muscle fibrosis, but this condition may be the result of fibrosis of the free wall beneath the papillary muscle, which impairs LV contraction. On CMR, patchy LGE may be seen in the papillary muscles. This enhancement may be focal, confined to the apical portions, or diffusely patchy, with sparing adjacent to the intramural coronary arteries.

### Infarction

Papillary muscle infarction occurs in 14% of all myocardial infarction cases and occurs more commonly in non-anterior distribution infarctions [59]. This results in mitral regurgitation [60] and ventricular arrhythmias [59]. Papillary muscle infarction is associated with a poor prognosis, including larger myocardial infarct size, worse LV function, a > 5-fold increase in the risk of major adverse cardiac events within the next 12 months, and a 4-fold increase in mortality [59].

**Fig. 15:**
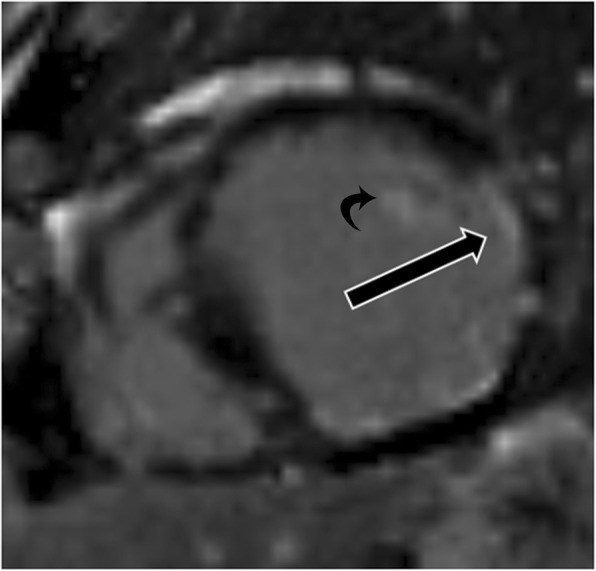
Papillary muscle infarction. Delayed enhancement image shows full thickness infarction of the anterolateral papillary muscle (curved arrow). There is also partial thickness scarring of the lateral ventricular wall due to myocardial infarction

On CMR images of papillary muscle infarction, LGE is seen in one or both of the papillary muscles (Fig. 15) [61], and associated infarction may also be seen in the ventricular myocardium. Papillary muscle contraction is limited (Additional file 13: Movie S13). In patients with a history of myocardial infarction, the presence of heterogeneous papillary muscle enhancement may be a harbinger of future arrhythmias [62]. CMR findings can be used to guide radiofrequency ablation of the arrhythmogenic foci in patients with arrhythmia, and mitral regurgitation can also be demonstrated and quantified with CMR.

### Rupture

Papillary muscle rupture can be iatrogenic or caused by infarction or trauma. It is a rare (0.5–5.0%) [5] but devastating sequela of papillary muscle infarction. Papillary muscle rupture is three times more common in the PM muscle, is the first coronary event in 80% [63], and may be seen with a small infarction in 50% [64]. Rupture typically occurs 2 to 7 days after the initial infarction and presents as acute mitral regurgitation and subsequent pulmonary edema and shock [65]. If left untreated, papillary muscle rupture has an 80% mortality rate and is responsible for approximately 5% of deaths after myocardial infarction [65]. The rupture may be total, which is usually fatal due to the total loss of mitral leaflet support, or partial involving some heads [66], in which survival depends on the extent of associated LV functional impairment. Even though only one head may be ruptured, the unruptured papillary muscle is also nearly always necrotic, contributing to mitral regurgitation [4]. Patients who require mitral valve surgery acutely after an inciting trauma tend to have complete ruptures [67].

CMR is not often used in the evaluation of patients with papillary muscle rupture since these patients are acutely ill and typically diagnosed through echocardiography [68]. On CMR, increased mobility of the mitral valve is suggestive of papillary muscle rupture [68]. There may also be abnormal LV wall motion related to the infarction [69].

## Masses and mass-like lesions

A number of masses and mass-like lesions can affect the papillary muscles. Although these entities may be benign and innocuous, a papillary muscle mass can sometimes be the sole cause of a patient’s symptoms. In the setting of a known malignancy, extra care should be taken in the evaluation of any abnormality to assess for possible metastatic disease.

### Calcification

**Fig. 16:**
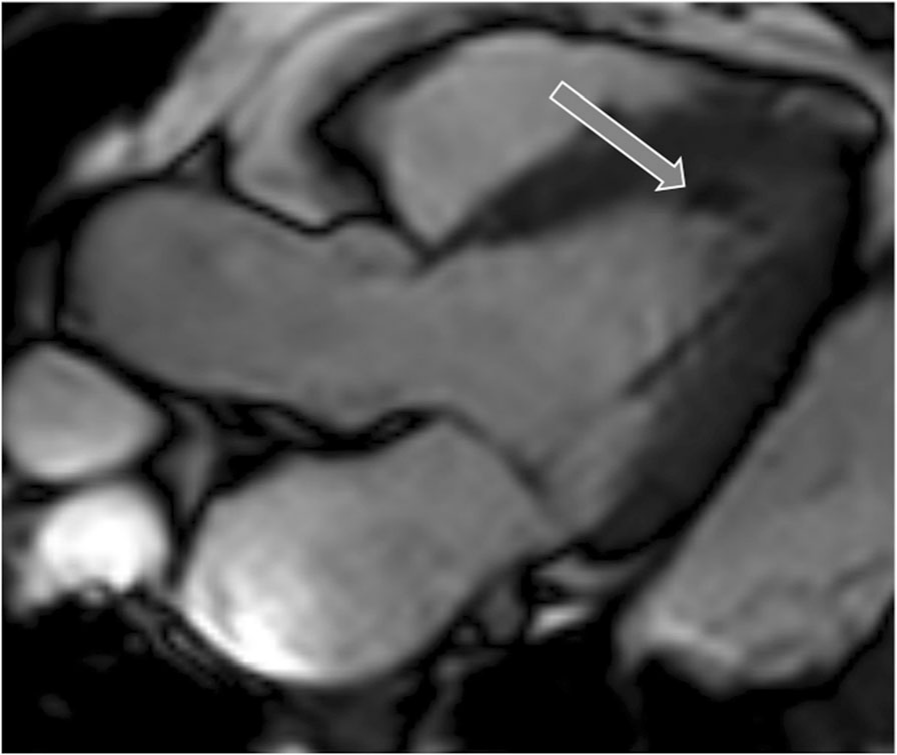
Calcification. Three-chamber LVOT view demonstrates hypointense signal at the apical portion of the anterolateral papillary muscle (arrow) related to calcification

Papillary muscle calcifications are noted in association with multiple entitites. In elderly patients, papillary calcifications in the apical region are within the normal spectrum (Fig. 16); however, diffuse and extensive calcifications are abnormal findings [70]. Pathological calcifications can be caused by intrinsic cardiovascular diseases such as coronary artery disease [70], infarction [71], dilated cardiomyopathy, and mitral valve disease or systemic diseases such as hypocalcemia or end-stage renal disease [70]. Calcifications in the fetus are normal in only 2% of cases [72]; 16% of cases are associated with trisomy 21 and 40% with trisomy 13 [72]. It is unclear what additional workup, if any, is merited for fetuses in which papillary muscle calcifications are incidentally identified. On CMR images, calcifications within the papillary muscles appear dark and may be best demonstrated on gradient echo imaging [73]. However, calcifications are typically better visualized on computed tomography.

### Thrombus and thrombus mimics

**Fig. 17:**
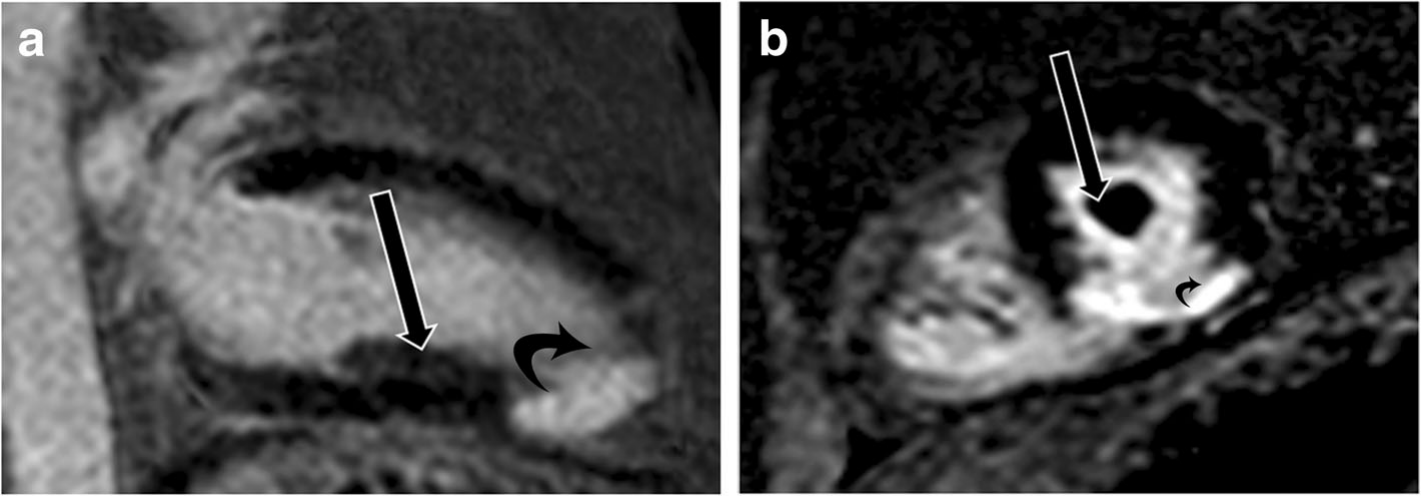
Thrombus. **a** Two-chamber vertical long axis delayed enhancement MRI image shows the thrombus (straight arrow) showing no contrast enhancement. Infarct is seen in the apical region (curved arrow). **b** Short-axis delayed enhancement image shows non-enhancing thrombus in the apical region (straight arrow) and apical infarct (curved arrow)

Thrombus, the most common non-neoplastic mass in the LV, can be located on the papillary muscle. Thrombus is commonly seen after myocardial infarction and is usually located in proximity to a region of systolic dysfunction (Additional file 14: Movie S14 and Additional file 15: Movie S15) [74]. Proper identification of ventricular thrombus is essential, as the initiation of anticoagulation therapy can significantly reduce the risk of embolic events [75]. On CMR, thrombi typically appear hypointense and avascular on all imaging sequences [76] (Fig. 17) with the exception of chronic vascularized thrombus, which may show some contrast enhancement. Thrombus remains dark even at long inversion times (e.g., 600 ms), unlike neoplasms. LGE MR images are highly sensitive, detecting thrombi of < 1 cm^3^ [75] and detecting an additional 50% of thrombi that cannot be detected on cine sequences [77].

**Fig. 18:**
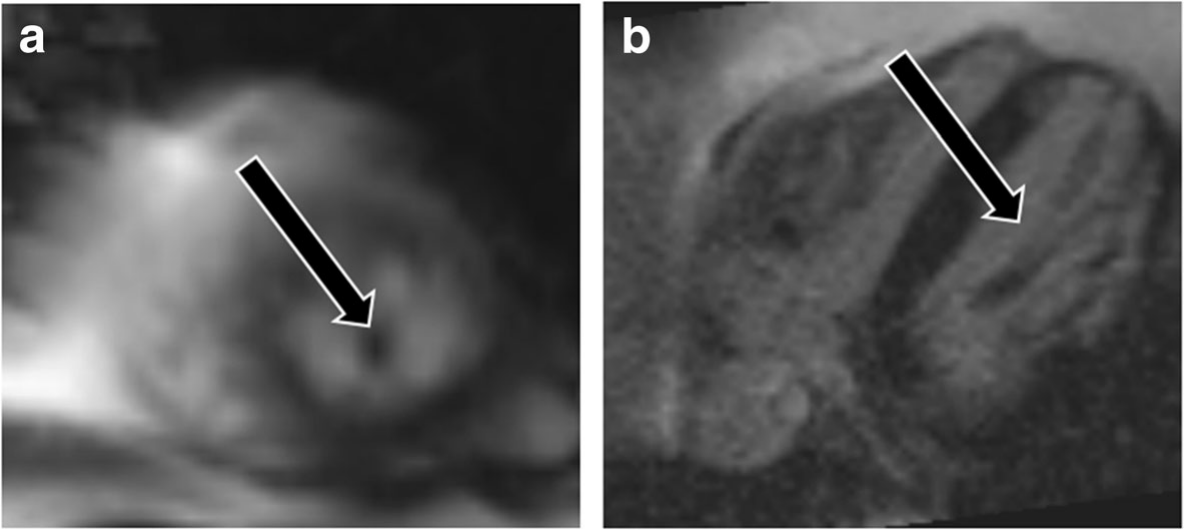
Accessory papillary muscle mimicking thrombus. **a** Short-axis SSFP shows hypointense region in the ventricular apex (arrow), thought to be thrombus on echocardiography. **b** Delayed-enhanced images with an inversion time > 600 ms show that the structure is not hypointense (arrow), consistent with an accessory papillary muscle rather than thrombus

Occasionally, a hypertrophied/variant papillary muscle may be mistaken for a thrombus, or a thrombus may be mistaken for an accessory papillary muscle [75]. With CMR, a thrombus remains dark on LGE images (both at regular and long inversion times), whereas a papillary muscle is isointense to the myocardium [76] (Additional file 16: Movie S16) (Fig. 18). The presence of an LV band is also helpful in identifying a papillary muscle [78].

### Benign neoplasms

**Fig. 19:**
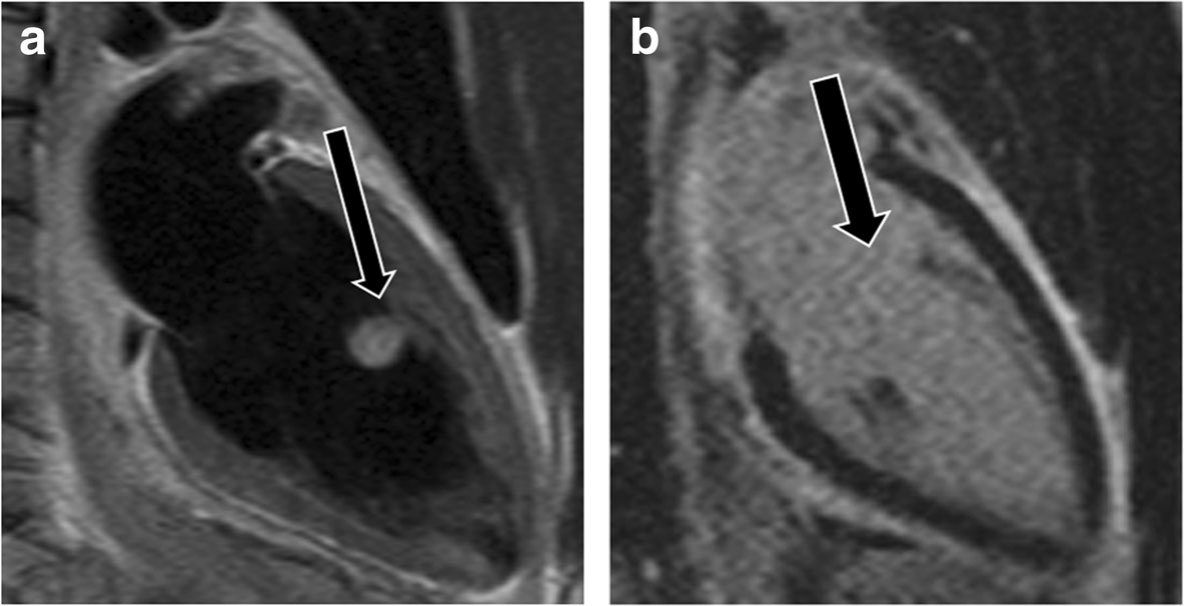
Myxoma. **a** Two-chamber T2-weighted dark blood image shows a hyperintense mass involving the anterolateral papillary muscle (arrow). **b** The mass enhances after contrast-enhancement (arrow) making it isointense to the blood pool. This was proven to be a myxoma

**Fig. 20:**
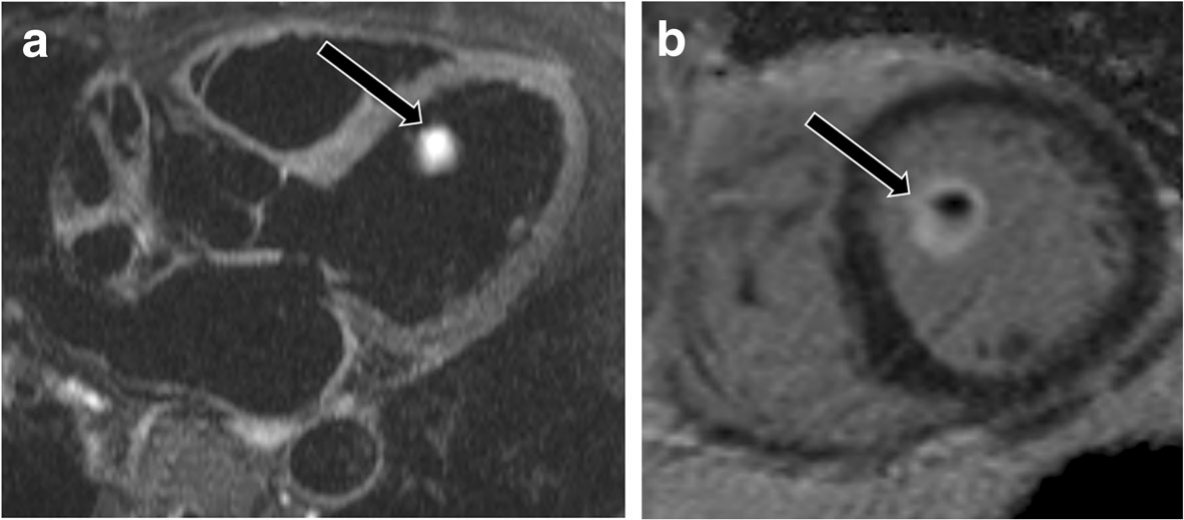
Fibroelastoma. **a** Three-chamber T2-weighted dark blood MR image shows a hyperintense mass attached to the anterolateral papillary muscle (arrow). **b** Short-axis delayed enhancement image shows delayed contrast enhancement (arrow). This was shown to be a fibroelastoma

**Fig. 21:**
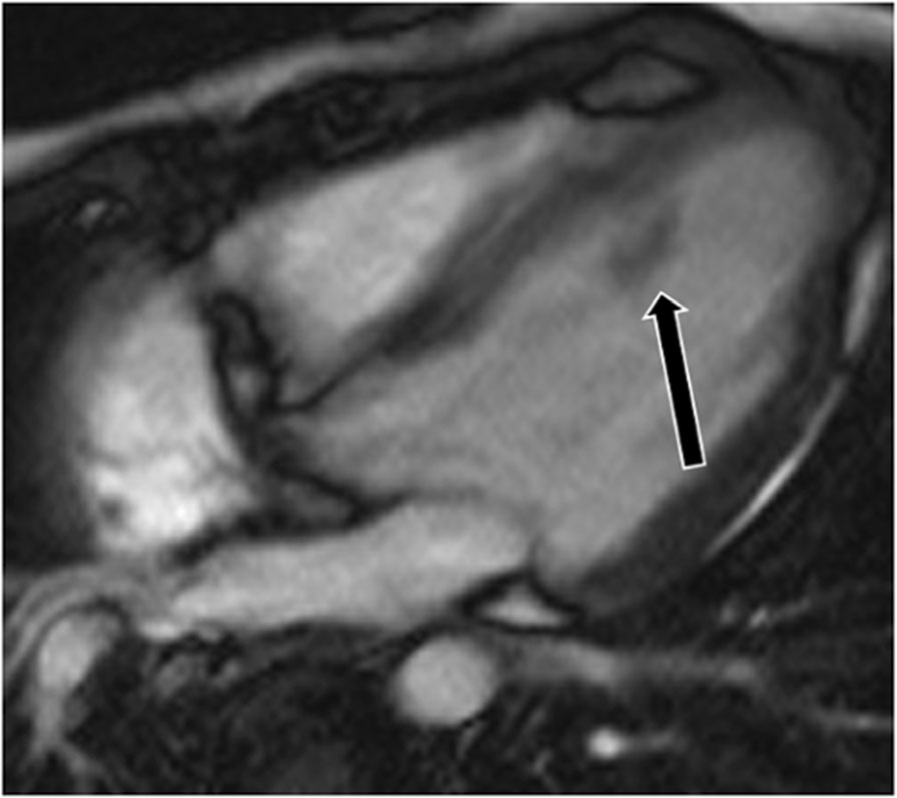
Rhabdomyoma. Four-chamber cine SSFP image shows a subtle mass isointense to myocardium attached to the papillary muscle (arrow). This was shown to be a rhabdomyoma. The patient also had a large subependymal nodule in the brain due to subependymal giant cell astrocytoma, related to the patient’s underlying tuberous sclerosis

Neoplasms involving the papillary muscles may present with symptoms related to obstruction from mass effect, systemic thromboembolic events, or arrhythmias [79]. Myxoma is the most common benign cardiac neoplasm. Although myxoma is usually seen in the left atrium, these neoplasms may involve the papillary muscles. A myxoma is typically lobular and mobile, with bright signal on T2-weighted images and heterogeneous LGE (Fig. 19, Additional file 17: Movie S17). Hemangioma is a vascular neoplasm that is lobular with a broad base [80]. On CMR, a hemangioma is well defined, is round or oval, has high signal on T2-weighted images, and shows contrast enhancement. Lipoma, another benign neoplasm, has high signal on T1-weighted images with signal dropout on fat-saturated sequences and no LGE [77]. Papillary fibroelastoma is most commonly seen in the valves but may originate from the papillary muscle [77]. This neoplasm has a stalk and is smooth. Papillary fibroelastoma has high signal on T2-weighted images and is isointense or slightly hyperintense on T1-weighted images with LGE (Fig. 20) [77]. Rhabdomyoma is the most common pediatric cardiac neoplasm and is associated with tuberous sclerosis. This neoplasm is smooth and broad-based and has signal similar to that of myocardium on all sequences (Fig. 21) [77]. Fibroma, the second most common pediatric cardiac tumor, is associated with Gardner’s syndrome. Fibroma is smooth, has low signal on T2-weighted images, and demonstrates intense LGE [77].

### Malignant neoplasms

**Fig. 22:**
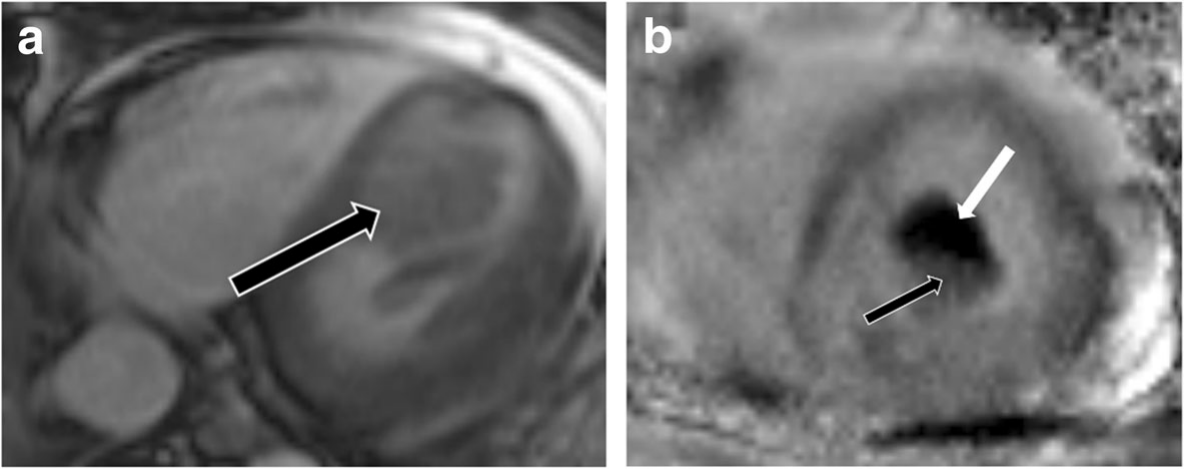
Metastasis. **a** Four-chamber SSFP image demonstrates a large hypointense mass of the anterolateral papillary muscle (arrows). **b** Short-axis delayed-enhancement image shows heterogeneous enhancement of the mass (black arrow). Attached to the mass is a markedly hypointense focus (white arrows) consistent with adherent thrombus. The patient had a history of metastatic thyroid cancer

**Fig. 23:**
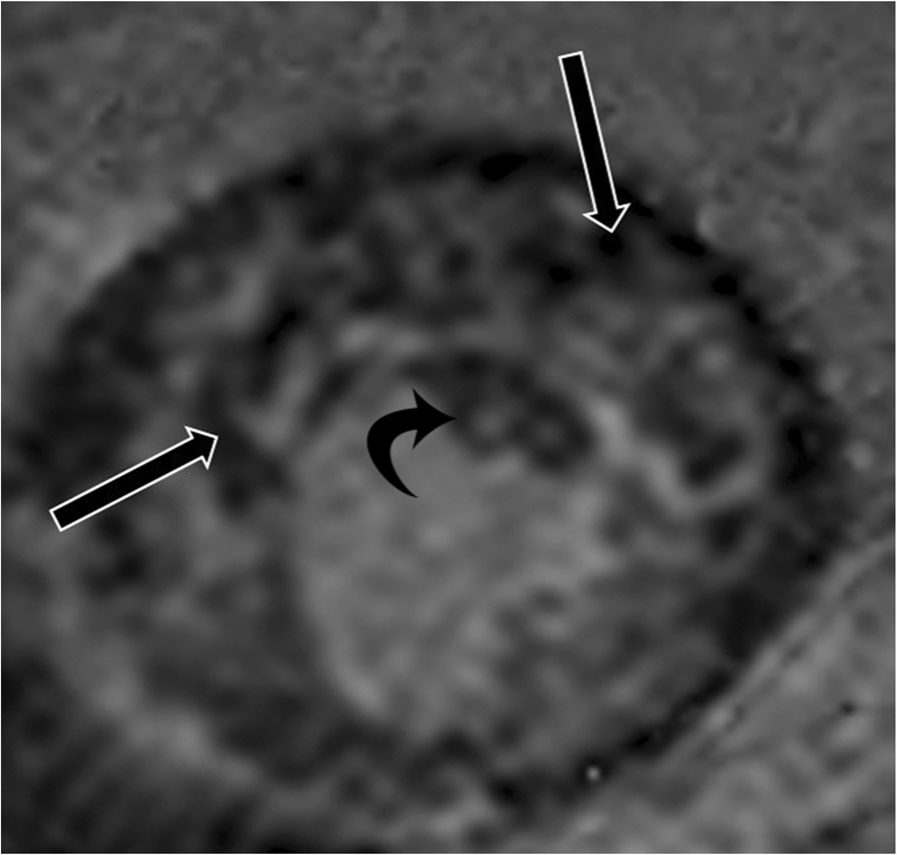
Leukemia. Short-axis delayed-enhanced image in a patient with leukemia demonstrates diffuse infiltration involving the papillary muscles (curved arrow) and myocardium (straight arrows)

Metastasis is the most common malignant neoplasm to involve the papillary muscles (Fig. 22, Additional file 18: Movie S18) [79]. Metastasis to the papillary muscles can occur via multiple pathways including hematogenous, lymphatic, and direct invasion, usually from melanoma, lung cancer, and breast cancer. Cardiac metastases can present as diffuse involvement or as discrete nodules [81]. Less than 1% of cardiac neoplasms are primary malignancies, and of the primary cardiac neoplasms, only 25% are malignant. Sarcoma is the most common primary cardiac malignancy [79]. The most common cardiac sarcoma is angiosarcoma, which presents as a lobular tumor with heterogeneous signal on T2-weighted images and heterogeneous enhancement [77]. Although angiosarcomas may involve the papillary muscles, they occur most commonly in the right atrium [82]. Other sarcomas include rhabdomyosarcoma, leiomyosarcoma, liposarcoma, osteosarcoma (which may calcify), malignant fibrous histiocytoma, and undifferentiated sarcoma [80]. Secondary lymphomatous involvement of the heart in the setting of widespread metastases is common, with 20% of autopsy cases demonstrating cardiac involvement [83]. Primary cardiac lymphoma is quite rare and is more often seen in immunocompromised patients [83]. CMR imaging in such cases shows isointense signal on T1- and T2-weighted images and heterogeneous enhancement. The involvement of multiple cardiac chambers is the rule rather than the exception [80]. On CMR images, leukemia presents as diffuse infiltration and contrast enhancement (Fig. 23).

## Conclusion

CMR is valuable in the evaluation of papillary muscle anatomy, function, and pathological abnormalities. CMR can demonstrate the wide variation in the morphology of papillary muscles, some of which can cause significant morbidity, particularly LV outflow obstruction. CMR is useful for distinguishing among thrombus, benign neoplasms, and malignant neoplasms.

## Additional files

Additional file 1: **Movie S1.** 2 chamber myocardial tagging MRI image shows deformation of tag lines though the anterior and inferior myocardial walls and papillary muscles in a normal person (MP4 840 kb)

Additional file 2: **Movie S2.** 4-chamber cine SSFP image in a patient with parachute-like asymmetric mitral valve shows asymmetric elongation of the anterolateral papillary muscle, with mild mitral regurgitation. (MP4 414 kb)

Additional file 3: **Movie S3.** 2-chamber cine SSFP image shows a band/septum extending horizontally across the LV dividing the LV into two chamber. The distal chamber has lower function. (MP4 340 kb)

Additional file 4: **Movie S4.** 4-chamber cine-SSFP sequence in the same patient as in Movie S3 shows the band/septum connecting the papillary muscles and dividing the LV into two chambers. The apical chamber is dilated and has lower systolic function. (MP4 345 kb)

Additional file 5: **Movie S5.** 3-chamber cine-SSFP image shows prominent trabeculations in the left ventricle, with the ratio of non-compacted to compacted myocardium of 9, consistent with LV non-compaction. There is also non-compaction involving the right ventricle. (AVI 1330 kb)

Additional file 6: **Movie S6.** 4-chamber cine SSFP image shows hypertrophied papillary muscles which cause obstruction at the level of mid-cavity with resultant flow acceleration seen in cine MRI image. This is more distal than the usual level of obstruction, which is at the LVOT. (MP4 705 kb)

Additional file 7: **Movie S7.** 4-chamber cine SSFP image shows anomalous insertion of papillary muscle to the mid portion of anterior mitral leaflet, which is producing LVOT obstruction, even in the absence of significant basal septal hypertrophy. (MP4 913 kb)

Additional file 8: **Movie S8.** 4-chamber cine SSFP sequence shows anomalous insertion of the anterolateral papillary muscle to the basal septum, instead of attachment to the mitral leaflet. (MP4 513 kb)

Additional file 9: **Movie S9.** Four-chamber SSFP shows only mild left ventricular hypertrophy, but the papillary muscles are hypermobile with a significant amount of slack. There is septal contact in systole, resulting in left ventricular outflow tract obstruction. (MP4 869 kb)

Additional file 10: **Movie S10.** 4-chamber cine SSFP image shows an elongated anterior mitral leaflet, which results in septal contact causing LVOT obstruction (MP4 869 kb)

Additional file 12: **Movie S12.** 4 chamber image shows the medial head of anterolateral papillary muscle attached to the mid portion of the anterior mitral leaflet. In addition, there is a thin accessory papillary muscle. There is also an eccentric mitral regurgitation. (MP4 829 kb)

Additional file 13: **Movie S13.** Short-axis cine SSFP images show infarction of the posteromedial papillary muscle, which is thin, irregular and shows restricted motion. (MP4 302 kb)

Additional file 14: **Movie S14.** Vertical long-axis cine SSFP showing hypointense thrombus at the anterolateral papillary muscle base. (MP4 878 kb)

Additional file 15: **Movie S15.** Four-chamber cine SSFP showing hypointense thrombus at the base of the anterolateral papillary muscle. (MP4 932 kb)

Additional file 16: **Movie S16.** Four-chamber cine SSFP shows a lesion thought to be a thrombus by echocardiography is actually an accessory papillary muscle that is attached to the LV apex. (MP4 2240 kb)

Additional file 17: **Movie S17.** Vertical long-axis cine SSFP image demonstrates an intermediate signal mass attached to the anterolateral papillary muscle. This was proven to be a myxoma. (MP4 769 kb)

Additional file 18: **Movie S18.** LVOT cine bright-blood images demonstrate a mass on the anterolateral papillary muscle in a patient with a history of widespread breast cancer. The mass can be seen extending to the LVOT and was found to be a metastatic deposit. (MP4 365 kb)

## Abbreviations

AL: Anterolateral
b-SSFP: Balanced steady-state free precession
CMR: Cardiovascular magnetic resonance
ECG: Electrocardiography
HCM: Hypertrophic cardiomyopathy
LAD: Left anterior descending artery
LCX: Left circumflex artery
LGE: Late gadolinium enhancement
LV: Left ventricle
LVOT: Left ventricular outflow tract
PM: Posteromedial
